# The Cryptographic Key Distribution System for IoT Systems in the MQTT Environment

**DOI:** 10.3390/s23115102

**Published:** 2023-05-26

**Authors:** Janusz Furtak

**Affiliations:** Faculty of Cybernetics, Military University of Technology, 00-908 Warsaw, Poland; janusz.furtak@wat.edu.pl; Tel.: +48-261-838-792

**Keywords:** key distribution system, cryptographic keys renewing, security in IoT, trusted platform module, MQTT secure data exchange

## Abstract

The Internet of Things (IoT) is a very abundant source of data, as well as a source of many vulnerabilities. A significant challenge is preparing security solutions to protect IoT nodes’ resources and the data exchanged. The difficulty usually stems from the insufficient resources of these nodes in terms of computing power, memory size, range energy resource, and wireless link performance. The paper presents the design and demonstrator of a system for symmetric cryptographic Key Generating, Renewing, and Distributing (KGRD). The system uses the TPM 2.0 hardware module to support cryptographic procedures, including creating trust structures, key generation, and securing the node’s exchange of data and resources. Clusters of sensor nodes and traditional systems can use the KGRD system to secure data exchange in the federated cooperation of systems with IoT-derived data sources. The transmission medium for exchanging data between KGRD system nodes is the Message Queuing Telemetry Transport (MQTT) service, which is commonly used in IoT networks.

## 1. Introduction

In an era of widespread data exchange, often from the very numerous sensor nodes of the Internet of Things (IoT), ensuring confidentiality and integrity is crucial. Cryptographic techniques, particularly asymmetric and symmetric key algorithms, are often used to protect data during storage and transmission. The properties of these algorithms determine their applications.

Asymmetric cryptography finds applications in establishing trust structures, signing transmitted messages and establishing a secure connection with another party for data exchange. With the use of asymmetric cryptography, it is possible to build solutions for the distribution of symmetric keys using the Diffie–Hellman scheme [1]. Asymmetric cryptography is unsuitable for encrypting large volumes of data because of the excessive overhead on the data to be encrypted, the consumption of large amounts of time and resources, and the need to use long keys to ensure adequate cypher strength.

The properties of symmetric cryptography are more favourable. They have lower computational power requirements and provide an adequate level of encryption power by using much shorter keys. Currently, the required encryption power forces the key of the asymmetric RSA algorithm to be no less than 3072 bits long and, for the symmetric AES algorithm, only 128 bits long [2]. In addition, symmetric algorithms provide good results when encrypting multimedia data streams [3]. A specific shortcoming of symmetric cryptography is the need for the secure distribution of the key to parties exchanging data. For this purpose, asymmetric cryptography establishes a secure connection between the parties involved. The first party generates a symmetric key (called a session key) and, using the established connection, transmits this key to the other party.

Every cryptographic key loses its validity. Strategies for determining the validity of keys vary. In some scenarios, the basis for determining the validity of a key is the volume of data encrypted using a given key. In other scenarios, the basis is the time since the key appeared. It is possible to use a combination of both strategies. Regardless of the strategy adopted, the need to renew keys arises. Key renewal strategies can also vary. Generating a new key using the same method as the previous one belongs to the first group of methods. In the second approach, the “old key” (before expiration) supports obtaining a “new key”. The third group includes methods in which two keys are generated initially, Where one will be the primary key, and the second will only support renewing the primary key when it expires.

The basis of cryptographic key distribution and renewal procedures is building trust between the parties involved. To build a trust structure, a so-called third-party trust is often required. In traditional solutions, the Certification Authority plays this role. Such a solution requires relatively large resources for storing cryptographic material, access to an efficient network connection, adequate computing power, and an efficient power source. For IoT network nodes, such a requirement is often impossible to meet [4,5].

For a network of sensor nodes, due to their limited capabilities, clusters are created. For such clusters, unique solutions are being prepared to ensure secure data exchange within the cluster [6,7]. Group key management (GKM) is used to distribute cryptographic keys for a group of cooperating nodes. An extensive analysis of GKM systems is presented in [8]. In this analysis, Dammak et al. separately evaluated implementations relating to wireless sensor networks (WSN) [9,10,11], wireless body area networks (WBAN) [12], wireless IPv6 networks [13], cloud computing [14], and IoT networks [15,16,17]. They considered the following properties of the solutions: key distribution schemes (centralised, distributed, decentralised), types of cryptographic algorithms used (asymmetric, symmetric, polynomial, attribute-based), key sharing by a group of nodes with a changing number of participants, scalability, the existence of single points of failure, mutual, and the independence of keys.

Solutions are needed to the problem of secure data exchange between clusters and other closed systems belonging, for example, to the critical infrastructure of the state, systems of the armed forces, police, health services, etc. In the case of a crisis caused by a natural disaster, terrorist events, or a war emergency, the need to ensure security cooperation will be crucial. Such cooperation includes integrating civilian IoT with military C2-class and logistics systems [18,19,20].

This paper describes symmetric Key Generating, Renewing, and Distributing (KGRD) for clients with limited memory, computing power, and energy resources, as well as for clients without such limitations. The KGRD system was developed based on the concept of this type of system described in [21]. The concept presented in [21] was not mature. The current study differs from that of [21] in how the solution concept differs from the implemented system. The study [21] was a presentation of the idea of solving the problem, while the current study is a description of the implemented system. During the work on implementing the KGRD system, many modifications were made to the data structures and the way data were secured, and the software TPM was used as a cryptographic coprocessor. Such modifications increased the number of procedures in the system and caused changes in the flow of individual procedures.

In the local trust structure of the hardware TPM, one asymmetric key was removed, and symmetric keys used to secure local data resources were introduced. The removed asymmetric key was originally used to authenticate newly registered nodes, so a new authentication method was developed using a unique NTAG tag and new tasks for node preparation and registration procedures. For the data stored in TPM NVRAM, access policies for this data have been implemented, raising the security level. Due to the use of the software TPM module as a cryptographic coprocessor, a simplified trust structure was implemented for the software TPM, and procedures for securely transferring symmetric keys were implemented from the hardware TPM to the software TPM. The most significant changes are related to the following aspects:

1. During the implementation, there was a problem with the hardware implementation of the commercially available TPM module. Several TPM hardware models tested (including the LetsTrust TPM and Optiga TPM) do not support encryption/decryption commands using symmetric keys. According to the [22] TCG specification, such modules should encrypt and decrypt. When developing the concept, I did not assume the existence of such a problem. The discovery of this problem made it necessary to change the concept of the whole system. I assumed that the hardware TPM would generate and store the keys, as well as secure the data stored in the hardware TPM’s resources and the node’s SD memory. A software TPM simulator will be used as a cryptographic coprocessor for symmetric encryption. This approach necessitated the secure transfer of symmetric keys, generated and stored in the hardware TPM, to the software TPM. I developed and implemented the procedure of duplicating keys and securely transferring the keys. The study refers to it in Section 3.5.
2. In the presented system, the operation of a node was modelled as a state machine. The statuses for each node were defined, a state transition diagram was developed, and all functions implementing the node’s state change were designed. I devoted Section 3.7 to describing the states of KS-type and N-type nodes.
3. Detailed descriptions of the implemented procedures are given in Section 3.8 and its subsections. There are three items that have expanded the list of implemented procedures compared to the concept. The names of the procedures are similar, but their contents are often entirely different.
4. A simplified way of handling the contexts of the data exchange protocol used between nodes in the system was introduced. The presented modifications forced minor changes to the file structures of the nodes stored in each node’s SD memory.
5. The implemented KGRD system has been tested. I evaluated the security level of the solutions used in the existing system. This is discussed in Section 3.9 and its subsections.

The essential elements of the contribution of the presented approach are as follows:

- a description of the modified solutions for the KGRD system designed to securely generate, distribute, and renew sensor nodes’ cryptographic keys;
- a description of a method of creating a trust for KGRD system nodes using a hardware TPM (to create local trust structures) and a software TPM as a crypto coprocessor;
- a description of the method of protecting the sensitive data of the system nodes;
- a description of the method of protecting sensitive data of nodes and the data exchange between system nodes;
- a description of how to use MQTT to transfer data among system nodes securely;
- an assessment of the solution’s resilience to the most common attacks on IoT networks;
- the implementation of the KGRD system demonstrator.

The rest of the article is organised as follows. Section 2 presents the general concept of the KGRD system. Section 3 describes the data structures and solutions used in the KGRD system. The security evaluation of these solutions also has its place in this chapter. Section 4 presents details of the hardware configuration, the system demonstrator’s implementation, and selected system test results. Discussion and planned work fill Section 5.

## 2. The General Concept of the Key Generation, Renewal, and Distribution (KGRD) System

### 2.1. The Idea behind the KGRD System

The number of IoT network nodes is proliferating. Any such node can be the target of a cyberattack, but this is a relatively rare situation. IoT nodes can often be part of a distributed tool to attack other targets. In both cases, the reason for this is the great difficulty in preparing appropriate mechanisms to protect IoT nodes from unwanted intrusion into node resources.

IoT nodes are often mobile, use short-range, low-bandwidth wireless links, have low computing power and memory, and have low-efficiency power sources. Due to these limitations, preparing effective security mechanisms to secure such nodes’ resources and ensure secure data exchange is a significant challenge. In such a situation, a standard solution is to create clusters of cooperating nodes. Security mechanisms are implemented to ensure the mutual authentication of cooperating nodes, the protection of nodes’ internal sensitive data, and the protection of mutually exchanged data. An example of such a solution is the secure domain of sensor nodes described in [6,7]. Such an approach solves the security problem within a group of sensor nodes, but secure data exchange between such clusters and other traditional information systems remains an open problem.

#### 2.1.1. Collaboration between Clusters of IoT Nodes and Other IT Systems

**Figure 1:**
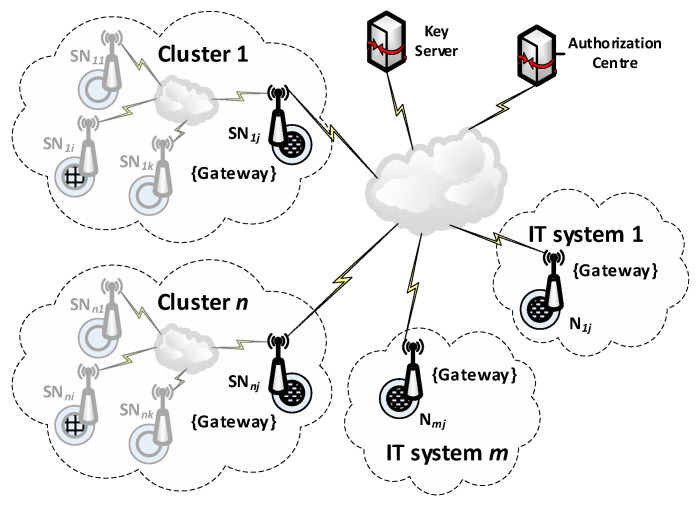
Illustration of IoT clusters and traditional information systems’ collaboration with the KGRD system (adapted from [21], taken from [23]).

Cryptographic solutions using symmetric key algorithms can be used to provide secure data exchange between clusters of IoT nodes. This approach has many advantages, but it requires a mechanism to distribute cryptographic keys safely. Cryptographic key distribution systems are known and effective for traditional IT systems. Still, there is a need to develop a cryptographic key distribution system that clusters of IoT nodes and traditional IT systems can use. Such demands meet the KGRD system. Figure 1 shows the structure of the KGRD system.

**Figure 2:**
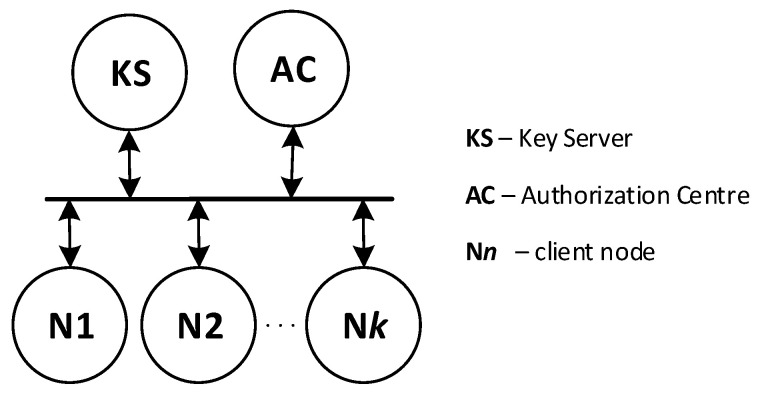
The structure of the KGRD system (taken from [23]).

It was assumed that, in each IoT cluster, one node would act as an intermediary for data exchange between its nodes and its environment. In Figure 1, such a node is labelled as Gateway. Nodes or software components of IT systems specially prepared for this task will play a similar role. For the KGRD system, Gateway components will represent the IoT cluster or IT system, respectively. Figure 2 shows a new look at the structure of the KGRD system.

#### 2.1.2. MQTT Service

**Figure 3:**
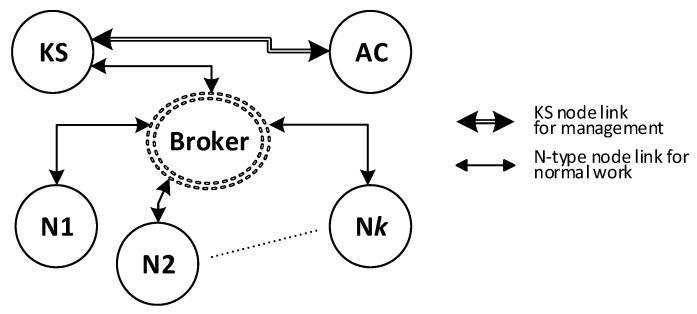
The structure of communication links in the KGRD system (taken from [23]).

IoT networks often use the MQTT service as an intermediary for data exchange. In the MQTT service, there are two types of nodes: a node that acts as a service server, called a broker, and other nodes that publish or subscribe to data. The intermediary of such an exchange is the service broker. Since the resources of IoT nodes are limited, it was assumed that the KGRD system would use the MQTT service to distribute keys. Figure 3 shows the KGRD system communication structure using the MQTT service.

#### 2.1.3. Components of the KGRD System

There will be four types of nodes in the KGRD system. These include:

- KS (Key Server)—the system’s most important node. Its main task will be to handle N-type nodes and, in particular, generating keys for symmetric cryptography and securely distributing keys to target nodes,
- AC (Authorization Centre)—the node will provide identifiers for authorised N-type nodes, and it will be the intermediary for the exchange of credentials necessary to register N-type nodes in the internal files of the KS node,
- N1, N2, … N*k* (Node)—nodes for which the KS node will provide keys,
- Broker—node necessary for the operation of the MQTT service, but in the KGRD system, it will only mediate the exchange of data between the other system nodes.

The KS node will only support N-type nodes registered earlier in the KS resource. The task of the AC node will be to provide authenticated data for the registration procedure of this node.

#### 2.1.4. The Idea of Acquiring a Key

**Figure 4:**
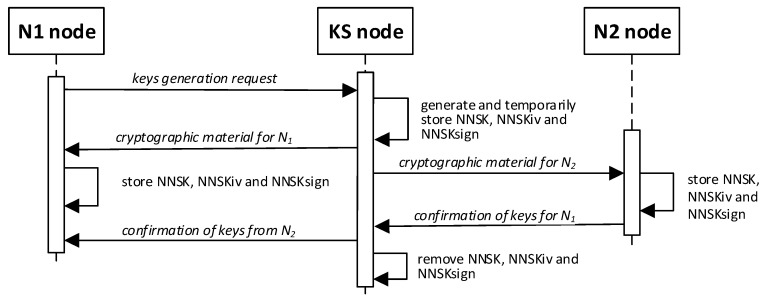
The key generation and distribution process for N1 and N2 nodes (taken from [21,23]).

A single key generation procedure will create two keys, named NNSK and NNSKsign, and an initialization vector named NNSKiv for a pair of N-type nodes. The NNSK (*Node-to-Node Security Key*) symmetric key and NNSKiv (*Node-to-Node Security Key Initialization Vector*) are designated to encrypt messages exchanged between Nm and Nn. The NNSKsign (*Node-to-Node Security Key for signing*) is assigned to determine the HMAC digest for the exchanged data [21,23]. Any registered N-type node can request node KS to generate keys for a pair of N-type nodes <N1; N2 >(N2 node must also be registered). Figure 4 shows the sequence diagram for the key generation and distribution procedure (it is assumed that, in all the figures, node N1 will send a request to generate keys for a pair of <N1, N2 > nodes).

## 3. Solutions in the KGRD System

### 3.1. Requirements for the KGRD System

Considering the conditions under which the cryptographic key distribution system is to operate and the limitations of IoT network nodes, the KGRD system should meet the following requirements [21,23]:

- KGRD generates symmetric keys only for authorised clients;
- the KGRD system works on the open Internet—KS and AC nodes of the system can build a trust relationship between each other using Certification Authority;
- the KS node will generate keys using a high entropy random number generator—this could be a quantum random number generator, for example;
- the AC node will be the source of authorised customer data for the KS node;
- the MQTT protocol will be used to exchange the data between system elements, especially during the cryptographic keys distributing procedure;
- the nodes of the system will cryptographically secure the stored data and data transmitted between the nodes of the system;
- each N-type node will have to be appropriately prepared and then registered in the KS node resources before it begins regular operation;
- the AC node will be responsible for providing the credential data to the N-type nodes necessary for the N node registration procedure;
- all cryptographic procedures of the KGRD system will be supported by a hardware TPM module (local trust structure creating, encryption/decryption, HMAC determination, key generating, etc.).

**Figure 5:**
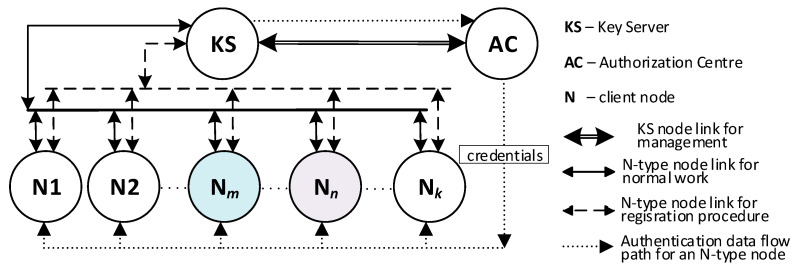
Communication channels in the KGRD system (taken from [21]).

Figure 5 shows how data are exchanged between elements of the KGRD system.

To properly organise message exchanges in the MQTT service, a particular string called “topic” is required, the contents of which must be known to the nodes that are the source of the message and to the nodes that are to be the recipients of the message. Before the message exchange begins, the receiving node must start subscribing to the “topic”, while the sending node will publish its messages using the “topic”.

Different types of messages will be sent between nodes in the KGRD system, so different “topic” strings will be designated for each system task. It was assumed, to improve security, that the contents of the “topic” strings would be generated randomly. These topics will only know a pair of nodes that will exchange data. In the following, these strings will be called TOPICn, where n = 0,1,2,3,4. Table 1 shows the list and use of each “topic” string.

### 3.2. Protection of the Resources of Each Node of the System

The system nodes have limited resources and the use of Certification Authority would be difficult, so it was assumed that all KS and N nodes would use a local trust structure. The KGRD system will use a hardware TPM v.2.0 module to create and support this trust structure [21]. The local trust structure would be used to build trust relationships between KGRD system nodes, protect the node’s sensitive data, and secure data exchange between system nodes.

The Trusted Computing Group has developed a cryptographic coprocessor standard called the TPM module [22]. It can be implemented as a hardware-on-chip or software-only TPM simulator. TPM supports the use of many cryptographic algorithms. These include SHA (256, 384, 512), HMAC, RSA (2048, 3072, 16384), ECC (256, 384, 521), and AES (128, 256) [22]. It also enables the generation of random numbers, the generation of asymmetric keys, the determination of hash functions, and the verification of digital signatures. The property that determines the usefulness of the TPM is the ability to store the private part of the asymmetric key in the internal structures of the chip. The TPM module generates the key only once, which is never accessible outside. The TPM places such keys on top of one of the cryptographic key hierarchies of the endorsement, platform, or storage hierarchy. This approach allows for the building of a local trust structure on each TPM-equipped node. Symmetric keys and the private part of asymmetric keys, residing at lower levels in the hierarchy, are protected by the key that is the parent of the key. These keys outside the TPM do not exist in explicit form. The TPM performs cryptographic operations using these keys internally. The TPM also includes NVRAM—where sensitive node data, such as cryptographic keys, can be securely stored—and a unique Platform Configuration Register (PCR), which allows the preparation of a mechanism for detecting unauthorised modifications to the node’s hardware and software configurations. In particular, PCR registers should be used in the system boot procedure to verify the correct hardware configuration of KS and N nodes and the integrity of critical system files. This procedure is not described in this paper.

KS and AC nodes will be implemented in an environment with no limitations regarding memory, computing power, and energy. Therefore, it will be possible to use Certification Authority capabilities to build a trust relationship between these nodes.

An asymmetric primary key will be at the top of the hierarchy in the local trust structure built based on the hardware TPM v.2.0 module (e.g., RSA2048 or ECC256). In the diagrams, it will be designated as SRK (Storage Root Key). The following key in the hierarchy will be the ANK (*Asymmetric Node Key*) [21,23], which will be used to protect the other keys the node uses.

### 3.3. KS Node Description

The most important node of the KGRD system is the KS node. The main tasks of this node will include generating symmetric keys and distributing these keys to the node that requested the keys, as well as to the node’s partner. The data necessary for execution can be grouped as follows:

- local trust structure;
- own node’s data;
- a description of the nodes authorised to be served by the KGRD system;
- temporary data containing generated cryptographic material for nodes in generating or renewing keys.

**Figure 6:**
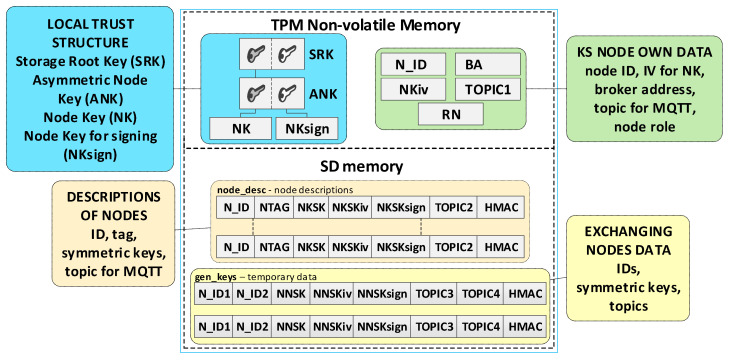
The data of the KS node stored in its resources (adapted from [21]).

The TPM will store the local trust structure and the own data of the node in its secure NVRAM. SD memory will store node descriptions and temporary data during key distribution. All data groups will be protected using the mechanisms offered by the TPM. Figure 6 shows how the KS node stores its sensitive data.

The KS node’s data include:

- SRK and ANK asymmetric keys (RSA2048), which are the beginning of the local trust structure;
- Keys—other elements of the local trust structure:
  ○ NK (Node Key) (AES128—16 bytes)—symmetric key for protecting the data in local SD memory;
  ○ NKsign (16 bytes)—key required to calculate HMAC for data encrypted with NK key;
- N_ID (4 bytes)—sensor node identifier in the KGRD system;
- BA (Broker Address)—IP address of MQTT broker;
- NKiv (16 bytes)—initialization vector for NK (random string);
- TOPIC1 (16 bytes)—N nodes use this topic to initiate key generation and distribution; this topic subscribes to the KS node; this topic is randomly generated and is known for all registered N nodes;
- RN (1 byte)—variable describing the node’s roles and status.

KS node files stored in its SD memory:

1. File **node_desc**—stores descriptions of N-type nodes that can use the KGRD system. There is one record in the file that includes a description of one node. Only the N_ID field in each file record is given in explicit form. The other fields are encrypted using the NK key. An HMAC is determined using the NKsign key for the N_ID field and the encrypted portion of the record. If only the N_ID and NTAG fields are filled in the record, it means that the node with the N_ID is not yet registered and cannot use the services of the KGRD system. Each file record includes the following fields (based on [21]):
  - N_ID (*Node ID*) (4 bytes)—the identifier for a node that is authorised to register in the system;
  - NTAG (*Node Tag*) (32 bytes)—field containing data to authenticate the node during its registry; SHA256 digest is determined from the concatenation of the N_ID field, the private part of the ANK key, and a 4 byte area containing the number of this node’s description entry in the node_desc file;
  - NKSK (*Node to Key server Security Key*) (16 bytes)—symmetric key for the encryption of data transmitted between the KS node and the registered N_ID node (node registration procedure generates this key–only the registered node and KS node know this key);
  - NKSKiv (16 bytes)—initialization vector for NKSK (random string);
  - NKSKsign (16 bytes)—key required to calculate HMAC for data encrypted with NKSK key (only the N_ID node and KS node know this key);
  - TOPIC2 (16 bytes)—N_ID node uses this topic (subscribes) to receive data from the KS node during key generation and distribution; this topic is randomly generated and is known for N_D and KS nodes;
  - HMAC–HMAC hash value is determined for the entire record using the NKsign key.
2. File **gen_keys**—the file store generated keys and topics from when these data were generated until the nodes for which these data were generated acknowledge receipts of these data. The identifiers, N_ID1 and N_ID2, are in plain text. The remaining areas are encrypted using the KS node’s NK key. HMAC is determined using the NKsign key for the N_ID1 and N_ID2 identifiers and the encrypted portion record. Each file record includes the following areas:
  - N_ID1 (4 bytes)—identifier of the node initiating key generation and distribution for nodes N_ID1 and N_ID2;
  - N_ID2 (4 bytes)—identifier of the second element in the pair;
  - NNSK (*Node to Node Security Key*) (16 bytes)—symmetric key for the encryption of data transmitted between nodes N_ID1 and N_ID2;
  - NNSKiv (16 bytes)—initialization vector for NNSK (random string);
  - NNSKsign (16 bytes)—key required to calculate HMAC for data encrypted with a NNSK key;
  - TOPIC3—the N_ID1 node uses this topic (subscribes) to receive data from node N_ID2; the N_ID2 node uses this topic (publishes) to send data from node N_ID1;
  - TOPIC4—the N_ID1 node uses this topic (publishes) to send data from node N_ID2; the N_ID2 node uses this topic (subscribes) to receive data from node N_ID1.

The KS node is the most crucial element of the KGRD system, so it should always be available to N nodes, have a permanent power supply, and use an adequate Internet connection. This node should be appropriately secured with a firewall, IDS/IPS systems, anti-virus software, etc. N-type nodes are likely to differ from each other and have limited capabilities. They are unlikely to have Ethernet or Wi-Fi connectivity, but they will use the communication links commonly used by IoT nodes. Therefore, the KS node should have a gateway for such communication links as ZigBee, Xbee, Thread, LoRa, BLE, etc.

### 3.4. N Node Description

N-type nodes are clients in the KGRD system. Each such node must have the data necessary to communicate with the KS node and secure the data transmitted between that node and the KS node. These data can be grouped as follows:

- N node local trust structure;
- own node’s data;
- data that are necessary to ensure the protection of data transmission between N nodes (identifiers, keys, initialization vectors, and “topic” strings).

**Figure 7:**
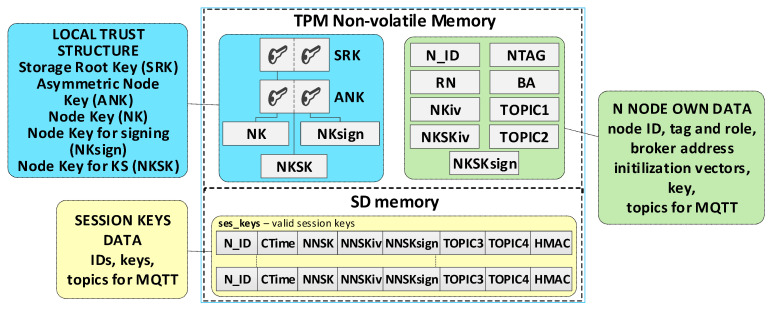
The data of the N node stored in its resources (adapted from [21]).

The node’s local trust structure and own data will be stored in the secure NVRAM of the TPM. The node’s SD memory will store cryptographic material to secure the data exchange. The node’s cryptographic material will be stored in the node’s SD memory, in encrypted form, using symmetric keys stored in a previously generated hierarchy of keys. All data groups will be protected using the mechanisms offered by the TPM. Figure 7 shows the way of storing data in the N node resources.

The N node’s data include:

- SRK and ANK asymmetric keys (RSA2048), which are the beginning of the node’s local trust structure;
- Keys—other elements of the local trust structure:
  ○ NK (*Node Key*) (AES128—16 bytes)—symmetric key for protecting the data in local SD memory;
  ○ NKsign (16 bytes)—key required to calculate HMAC for data encrypted with the NK key;
  ○ NKSK (*Node to Key server Security Key*) (AES128—16 bytes)—symmetric key for the encryption of data transmitted between the node and KS node (only the node and KS node know this key);
- NTAG (*Node Tag*) (32 bytes)—field containing data to authenticate the node during its registry; AC node passes this tag to the node when initializing the node in the KGRD system;
- N_ID (4 bytes)—identifier of the sensor node; passed by the AC node;
- RN (1 byte)—variable describing the node’s roles and status;
- BA (*Broker Address*)—IP address of MQTT broker;
- NKiv (16 bytes)—initialization vector for NK (random string);
- NKSKiv (16 bytes)—initialization vector for NKSK (random string);
- TOPIC1 (16 bytes)—the node uses this topic (publishes) to initiate the generation and distribution of symmetric keys (KS node subscribes to this topic);
- TOPIC2—the node subscribes to this topic while exchanging data with the node KS, while generating and distributing symmetric keys.

The **ses_keys** file is stored in the node’s SD memory. In this file, the node stores the cryptographic material necessary to secure the data exchange between this node and the nodes whose identifiers are given in the individual records of this file. A zeroed CTime field indicates that the cryptographic material has not yet been generated or has expired. The N_ID and CTime fields are in explicit form. The other areas are protected cryptographically with the NK key. HMAC is determined using the NKsign key for the public (N_ID and CTime) and encrypted record portions. Each file record includes the following areas:

- N_ID (4 bytes)—identifier of the second node in the pair with which the data exchange will be secured using cryptographic material stored in this record;
- CTime (*Creation Time of the key*) (8 bytes)—timestamp of the moment the cryptographic material is received from the KS node;
- NNSK (*Node to Node Security Key*) (16 bytes)—symmetric key for the encryption of data transmitted between the local node and node N_ID (known only for the local node and node N_ID);
- NNSKiv (16 bytes)—initialization vector for NNSK (random string);
- NNSKsign (*Node to Node Security Key for signing*) (16 bytes)—key required to calculate HMAC for the data encrypted with the NNSK key;
- TOPIC3—the local node uses this topic (subscribes) to receive data from node N_ID; the N_ID node uses this topic (publishes) to send data to the local node;
- TOPIC4—the local node uses the topic to send (publishes) data to node N_ID; the N_ID node uses this topic (subscribes) to receive data from the local node;

### 3.5. Ways of Implementing the N Node

The assumptions for the KGRD system did not specify large requirements for N-type nodes. Type nodes can either be implemented as standalone devices to support clusters of IoT network nodes or as software packages designed to support traditional IT systems. In the case of autonomous devices for IoT, it was assumed that these nodes can have limited memory, computing power, and energy resources, and they can be mobile and use wireless links. An example of such an implementation would be a Raspberry Pi board with a TPM v.2.0 hardware module installed. Depending on the demand and the TPM model used, N-type nodes can be implemented in one of the following ways:

- a standalone device with hardware TPM v.2.0 that meets all TCG specifications [22];
- a standalone device with a hardware TPM that does not meet all the requirements of the TCG specification [22] and, in particular, does not support encryption/decryption functions (such hardware TPM modules are available on the market, e.g., Infineon Iridium SLx 9670 and LetsTrust TPM 2.0);
- a standalone device using a software-based TPM simulator that has implemented all the functions described in the TCG specification [22];
- a software component that does not require a TPM fully emulates an N-type node’s operation. This component can obtain keys from the KGRD system for a traditional IT system to work with other N-type nodes.

The solutions for the KGRD system presented in this paper are prepared for an implementation case that uses a TPM module that does not support encryption/decryption functions. For this reason, the N node will use a software TPM simulator and the hardware TPM. The hardware TPM, except encryption/decryption functions, will perform all other security-related activities, particularly the secure storage of cryptographic keys and other sensitive N node data, random number generation, as well as SHA and HMAC digest determination.

**Figure 8:**
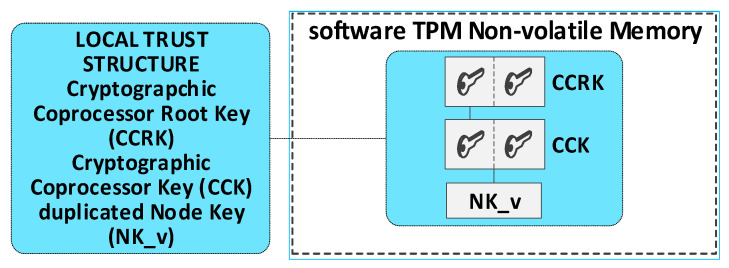
The key hierarchy in a software TPM after importing an NK key from a hardware TPM.

Before performing the encryption/decryption function, the key must be read from the hardware TPM’s resources and transferred to the keys hierarchy in the software TPM (this activity is performed once after powering up the Node N). For this purpose, a particular procedure is used to secure duplication of the transferred key on the hardware TPM side (the final section provides a detailed description of key duplication). Then, the importing of this duplicated key on the software TPM side is performed. Before starting the key duplication procedure in the software TPM, a simplified local trust structure must be generated, into which the duplicated key will be imported. The key hierarchies that are generated in the software TPM are not permanent (they disappear after powering down the N node), so a new local trust structure must be generated in the software TPM every time the node is powered down. Then, the transfer of the necessary keys, from the hardware TPM to the hierarchy in the software TPM, must be carried out. Figure 8 shows an example of the contents of the key hierarchy in a software TPM after importing an NK key from a hardware TPM.

The procedure for secure duplication is described in the TCG specification of the TPM module [22]. There are two TPM modules involved in key duplication. The first module is the hardware TPM—it will be labelled S1. The second module is the software module (S2). The duplication procedure includes the following steps:

1. Obtain the public part of the key from the S2 system, which is to be the new parent of the duplicated key (for example, CCK), and transfer it to the S1 system.
2. Execute the ANK key duplication command in the hardware TPM of the S1 system using the public part of the SRK key of the S2 system. The result is three files containing: the private part of the ANK key and the ANK key seed (both secured by the public key of the SRK system S2), as well as the public part of the ANK key.
3. Execute a key duplication command (e.g., NK) by the S1 module using the public part of the key from the S2 module (e.g., CCK). The result is three files containing: the private part of the duplicated key (NK) and the seed of that key (both bound to the public key CCK of the S2 system), as well as the public part of the duplicated key. These files require no other security and can be transferred to the S2 system.
4. Import the contents of the transferred files into the key hierarchy in the S2 system, starting with the new parent of the duplicate key (as an NK_v key).

### 3.6. AC Node Description

Among the assumptions of the KGRD system, one is to support only authorized N-type nodes. This assumption results in the fact that, before a KS node can perform its services for N nodes, both types of nodes will need to have the appropriate data. The KS node requires data about the N-type nodes authorised to use its services, while the N nodes will need credentials to initiate cooperation with the KS node. The AC node head tasks are all the activities necessary for data preparation for KS and N nodes.

KGRD system clients (N nodes) can vary widely. The differences may relate to the technology used, the way the system is protected, the purpose of the system, the classification of the processed data, national or organizational affiliation, etc. For this reason, it isn’t easy to define a homogeneous way to initiate an interaction between these systems and the KS node.

The solution to these problems is not the subject of this paper. Still, it has been assumed that the AC node will support the organizational activities required to establish the cooperation of such systems with the KS node. The AC node will be the intermediary for the secure transfer of {N_ID, NTAG} credentials from the KS node to the N nodes authorised to use the services of the KGRD system. As a result of these actions, the KS node will have a list of authorised N nodes, and the authorised N nodes will have their N_IDs, NTAGs, and the N_IDs of the nodes with which they can interact.

The target implementation of the AC node should meet the following requirements:

1. The configuration of the AC node must comply with the security recommendations for nodes operating on the Internet (an example of such requirements is given in the final section of Section 3.8).
2. Data exchange between the KS and AC node must be secured using mechanisms that use a recognised Certification Authority.
3. The KS node will prepare credentials for new N nodes.
4. The credentials for the N node will be transmitted, via a secure link, from the KS node to the AC node in a form that ensures the confidentiality and integrity of the data. The data packet will contain, among other things, an identifier for the node (N_ID) and a specially prepared NTAG tag. This tag is prepared so that it can be used only by the node for which it was prepared to register only with the KS node that prepared this tag.
5. The credentials will be transmitted to the N node in a protected and controlled environment using a secure link, such as an SSH service.

### 3.7. Status of Nodes and Activities in the KGRD System

**Figure 9:**
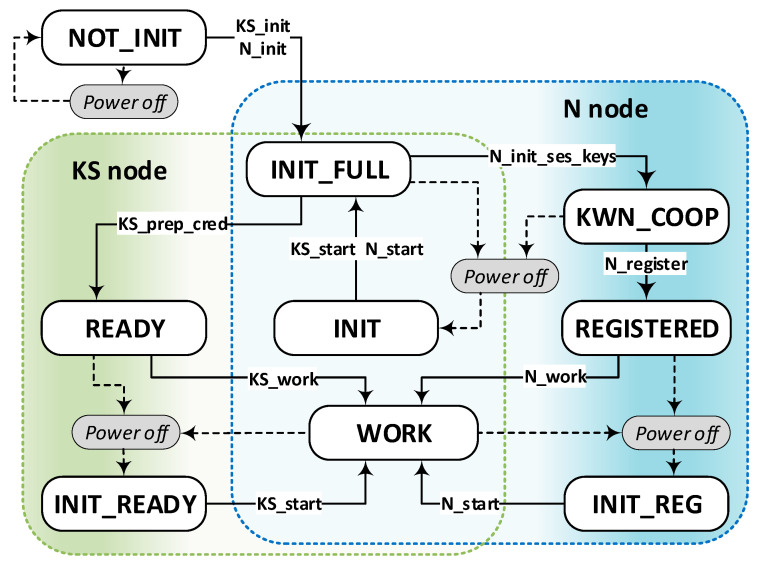
System node states and state change procedures.

During its lifetime, each node can be in one of the states. Table 2 describes the states of the KS and N nodes. Figure 9 illustrates how to transition between states. This figure also gives the names of the procedures that make the state change possible.

### 3.8. Procedures in the KGRD System

Building a secure system requires the development of procedures that will be used throughout the system’s life, i.e., from the moment of its creation through the processes of its operation and, finally, its decommissioning. These procedures will implement security solutions for hardware and software configuration. The list of these procedures (based on [21]) is as follows (in parentheses are the names of the implemented functions):

1. The procedure for starting the Broker node.
2. **The procedure for initiating the KS node (KS_init)**.
3. The procedure for preparing the credentials for an N node (**KS_prep_cred**).
4. The procedure for establishing a cooperation KS node with the Broker node (**KS_work**).
5. **The procedure for initiating the N node (N_init)**.
6. The procedure for setting the list of cooperators for node N (**N_init_ses_keys**).
7. **The procedure for registration of the N node in the KGRD system (N_register)**.
8. The procedure for establishing a cooperation N node with the Broker node (**N_work**).
9. **The procedure for generating and distributing symmetric keys (N_request_key)** involves three steps:
  (a) request a set of cryptographic keys and topics;
  (b) provide the generated cryptographic material to the destination node;
  (c) confirm the transfer of cryptographic material.
10. **Procedure for secure data exchange between nodes (N_data_send).**
11. The procedure for renewing and distributing a symmetric key involves three steps:
  (a) request the renewal of cryptographic material—includes activities such as notifying the other party of the initiation of the procedure,
  (b) deliver renewed cryptographic material to the nodes concerned,
  (c) confirm the transfer of cryptographic material.
12. The procedure for restarting the node after powering on again (**KS_start**, **N_start**).

The procedures highlighted in bold in the above list are described in detail in the following subsections.

Each node of the KGRD system should be secured under good security practices. In particular, the following should be done:

1. Update the system firmware.
2. Remove default installed and unnecessary accounts from the node’s operating system (e.g., in Raspbian, remove the account named “pi”) and change the passwords of the remaining accounts to something other than the default (as required by the security policy).
3. Create another account that will be dedicated to the remote management of the node acting as a Broker. Logging into this account must require a password. This account can have the superuser privilege (to use “sudo” command) but with the superuser account password enforced.
4. Run an SSH service that allows remote login via a secure link. The SSH service should be configured so that only selected accounts can be remotely logged in.
5. Run and configure the MQTT service to force the use of TLS protocol (port tcp/8883). This approach requires certificates for the broker and the broker’s clients. A way to obtain such certificates is to use a self-signed Certificate Authority and server-side certificates on the Broker node. The generated certificates for the clients can be forwarded to the N nodes, via the AC node, as they pass their credentials to the nodes.
6. Run and configure the local firewall (e.g., UFW) so that the node only supports the ports required for operation:
  - to support SSH (port tcp/22) and, additionally, block traffic for six or more post-connection attempts from the same IP address in the last 30 s (to prevent brute force attacks),
  - to support MQTT over a secured link (port tcp/8883)
7. Disable all services that are not needed for broker operation in the system.
8. Disable all unnecessary interfaces of the system, e.g., Bluetooth, Serial ports.

#### 3.8.1. The Procedure for Starting the Broker Node

The node that plays the Broker role should be run first in the system. This node only acts as an intermediary for data exchange between other nodes in the KGRD system, but it should be configured under good security practices and should accept all requests from other nodes in the KGRD system described in the following subsections. The Broker node configuration must meet the requirement described in the final section of Section 3.8.

#### 3.8.2. The Procedure for Initiating the KS Node

Initializing the KS node is the next mandatory step after launching the Broker node. This procedure performs the following tasks:

1. the generation of trust structure in hardware TPM, including SRK and ANK keys;
2. the generation of symmetric keys, NK and NKsign, and attaching them to the created trust structure—these keys and the NKiv field will be used to secure the node’s resources stored in SD memory;
3. the generation of a temporary trust structure in a software TPM, including CCRK and CCK keys;
4. the duplication of the NK key from the hardware TPM module and securely importing this key (NK_v) into the trust structure in the software TPM module;
5. the creation and initialization of the following areas in the NVRAM of the hardware TPM: N_ID, BA, TOPIC1, NKiv, and RN;
6. set the status for the KS node to “INIT_FULL” in the RN field.

**Figure 10:**
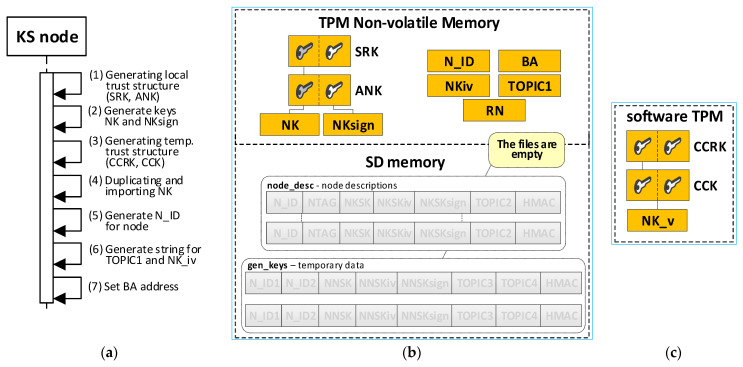
Sequence diagram: (**a**) the KS node’s resource contents in the hardware TPM (**b**) and software TPM (**c**) after the KS node initialization procedure (adapted from [21]).

Figure 10 shows the sequence diagram and the KS node resource contents after the procedure is completed (updated fields are highlighted in orange).

#### 3.8.3. The Procedure for Preparing the Credentials for the N Node

The procedure is designed to generate credentials for N nodes in the KS node resources. These data are placed in the “node_desc” file in SD memory.

The operation of an AC node in the system will depend very much on where the KGRD system is applied for this study, so it was assumed that the procedure described here would additionally export the generated authentication data to a separate file with the working name “node_desc_export”. This file will be the source of authentication data for N nodes in the procedure for initiating these nodes. How to use this file is shown in Section 3.8.5.

**Figure 11:**
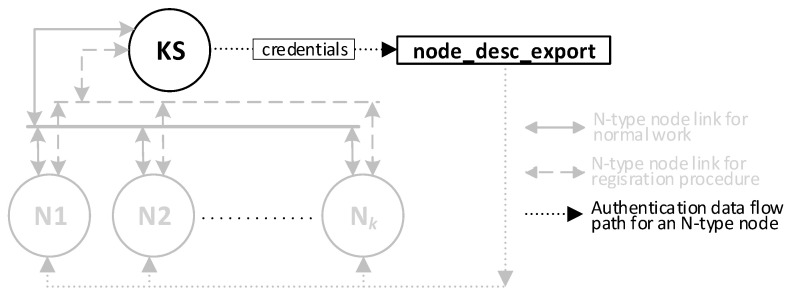
Transfer file “node_desc_export” (adapted from [21]).

**Figure 12:**
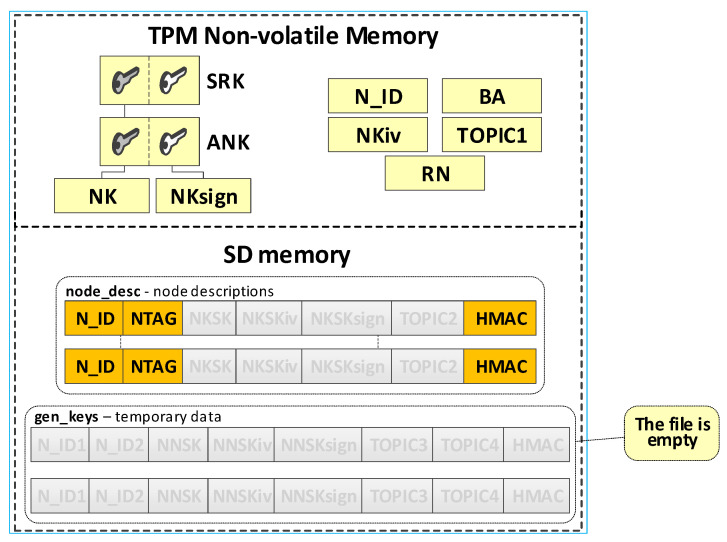
KS node data resources after the credentials preparation procedure is completed (adapted from [21]).

The result of the procedure for generating credentials will be two files. The first file is the “node_desc”, which is stored in the KS node resources. Figure 11 shows how this file is transferred in the KGRD system. Figure 12 shows the contents of this file after generating the credentials for N-type nodes (the data updated so far are highlighted in yellow, and those updated in the last step are in orange). The second file is “node_desc_export”. 

After completing this procedure, the status of the KS node is set to “READY” in the RN variable.

#### 3.8.4. Establishing Cooperation KS node with the Broker Node

The procedure starts the cooperation between KS nodes and the Broker, as well as subscription in the “TOPIC0” and “TOPIC1” topics.

After completing this procedure, the KS node status is set to “WORK” in the RN variable.

#### 3.8.5. The N Node Initiation Procedure

The procedure for initializing an N-type node is to prepare the node for operation in the KGRD system. This procedure generates a local trust structure in the node’s hardware TPM that includes SRK and ANK keys. It then generates NK and NKsign keys for this node and attaches them to the generated trust structure.

In the next step, a trust structure is created in the software TPM, and a duplicate NK key is imported into this structure using a particular and secure procedure.

**Figure 13:**
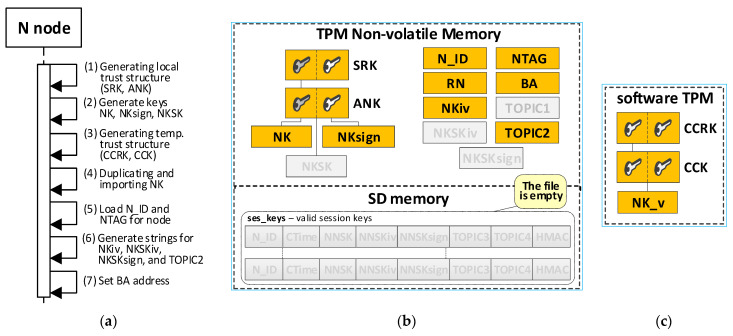
Sequence diagram: (**a**) the N node’s resources contents in the hardware TPM (**b**) and software TPM (**c**) after the N node initialization procedure (adapted from [21]).

**Figure 14:**
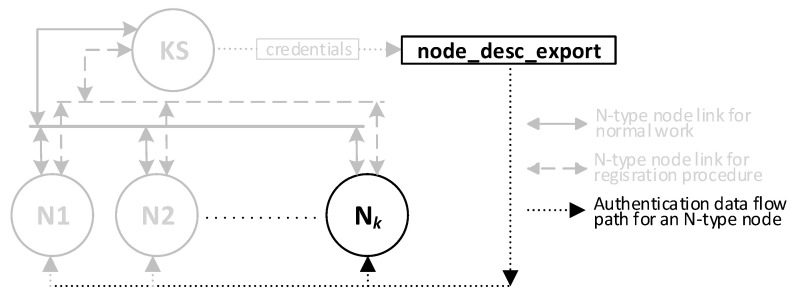
Data transfer from the “node_descr_export” file during the N*_k_* node initialization procedure (adapted from [21]).

The last step generates the fields necessary for an N-type node, including NTAG, N_ID, BA, NKiv, NKSKiv, NKSKsign, TOPIC2, and RN. NTAG, and N_ID fields are generated based on the provided “node_desc_export” file. The Broker node IP address is placed in the BA field. The contents of the fields, NKiv and TOPIC2, are generated randomly. The last action of this procedure is to set the status value for the node to “INIT_FULL” in the RN field. Figure 13 shows the contents of this file after generating the credentials for N-type nodes (so far, updated data is highlighted in orange). Figure 14 shows how the data from this file is transferred to the initiated node.

#### 3.8.6. Procedure for Setting the List of Cooperators for Node N

**Figure 15:**
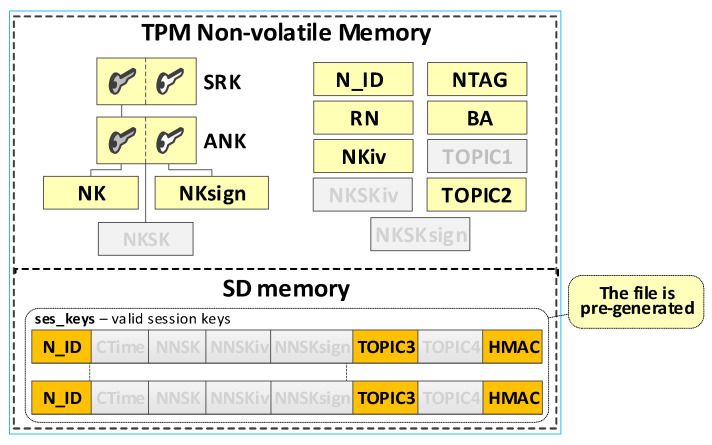
N node data resources after setting the list of cooperators procedure is completed (adapted from [21]).

In the target implementation of the KGRD system for N-type nodes, the source of messages about other N-type nodes that can securely exchange data with a given N-type node will be the AC node. The described solution assumes that all N-type nodes known to the KS node and registered in the KS resource can exchange data. Each registered N-type node can request the KS server to generate cryptographic material to cooperate with each registered N-type node. The source of the data will also be the “node_desc_export” file. This procedure will result in a pre-generated file “ses_keys”. Figure 15 shows the resources of node N after completing this procedure (the data updated so far are highlighted in yellow, and those updated in the last step are in orange).

After completing this procedure, the N node status is set to “KWN_COOP” in the RN variable.

#### 3.8.7. The Registration Procedure for the N Node in the KGRD System

This procedure is designed to have node N registered in the KS node’s resources and transfer NKSK and NKSKSign keys, as well as the NKiv vector, to the KS node to protect future data exchanges between the KS and N nodes in question.

At first, node N subscribes to the “TOPIC2” topic on the MQTT service. The node’s N_ID and NTAG, which the node acquired from the “node_desc_export” file in this node’s initialization procedure, and the “TOPIC2” compose the payload of the ***nksk_key_req*** request frame. The payload of this frame is encrypted with NTAG, and it is treated as a one-time use password. An HMAC digest is calculated using the NTAG field as the key for the entire frame. The ***nksk_key_req*** frame constructed in this way is published to the KS node. The KS node verifies the correctness of the received frame and ignores the request if it detects any irregularities. These irregularities include an invalid HMAC, a KS “node_desc” file lacking a description of the node that issued the request, or an invalid NTAG.

**Figure 16:**
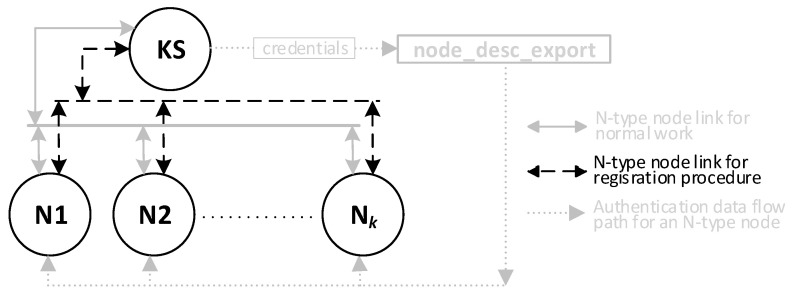
The way N-type nodes interact with KS nodes during the procedure. N-type nodes’ registration procedure (adapted from [21]).

**Figure 17:**
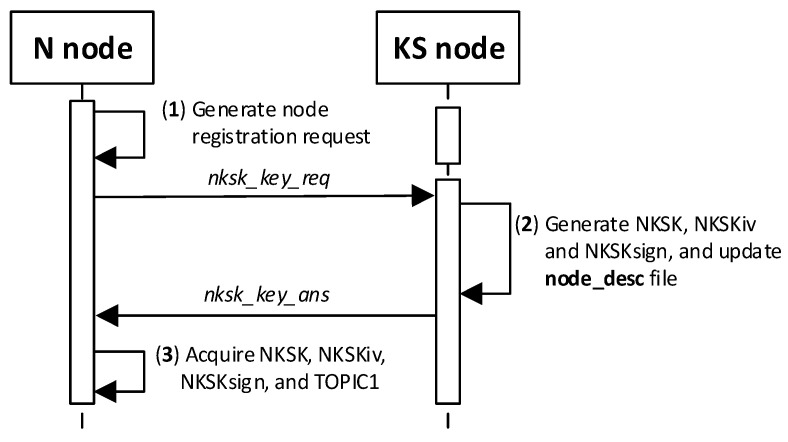
The sequence diagram for the registration procedure of N-type nodes (taken from [21]).

The KS node then updates the description of that node in the local node_desc file and sends the contents of its TOPIC1 field to the registered node in response. The fields received in the request frame are used to secure the response frame: the NTAG encrypts the frame payload and calculates the HMAC digest for the response frame. Figure 16 shows how N-type nodes interact with KS nodes during the procedure. Figure 17 shows the sequence diagram for registering an N-type node.

**Figure 18:**
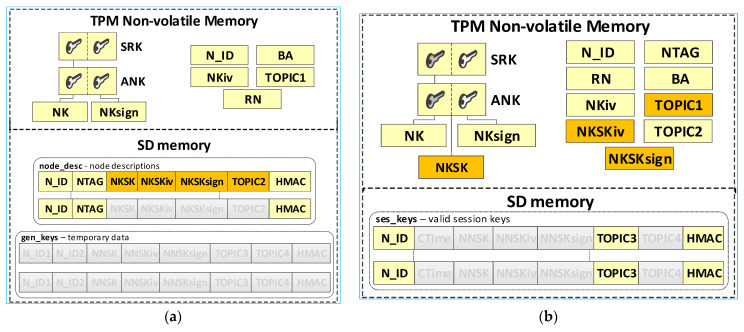
The KS node’s data resources after registering the first N-type node (**a**) and the N-type node data resources after registering it (**b**) (adapted from [21]).

The result of the registration procedure of the N-type node is an update of the KS node “node_desc” file and the TPM memory of the registered node file. Figure 18 shows the KS node resources after the first N node is registered and the resources of the first N node after it is registered (the data updated so far are highlighted in yellow, and the updated data after the registration procedure are highlighted in orange).

After completing this procedure, the N node status is set to “REGISTERED” in the RN variable.

**Figure 19:**
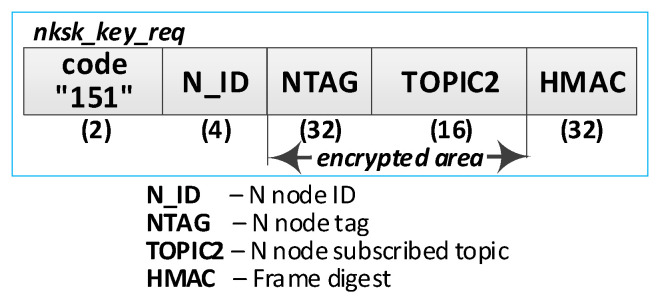
The node registration request frame (adapted from [21]).

**Figure 20:**
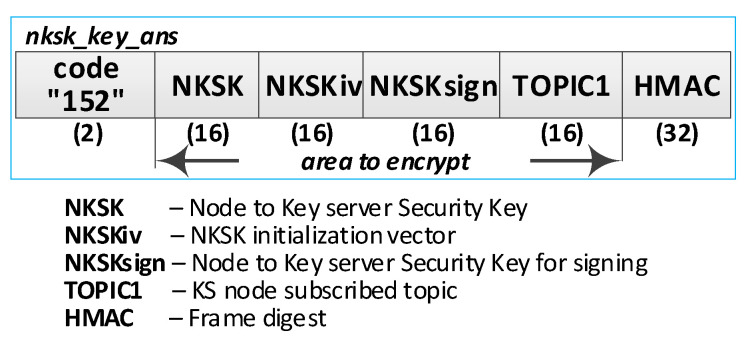
Node registration confirmation frame (adapted from [21]).

The following are the descriptions of the most critical stages of the procedure (the numbers in brackets (e.g., (1)) before the stage name corresponds to the designation of that stage in Figure 17):

(1) **Generate node registration request**—a node sending a registration request to a KS node initiates the authentication process of that node in the system and expects to send NKSK and NKSKsign keys, NKSKiv, and TOPIC1 strings. Activities to be performed:
  - The N node builds the ***nksk_key_req*** frame (Figure 19). The frame payload is encrypted, and the HMAC digest is calculated. Both of these protection actions use the NTAG field as a key.
  - The N node sends the ***nksk_key_req*** frame using the TOPIC0 topic.
(2) **Generate cryptographic material:** generate NKSK and NKSKsign keys, the NKSKiv initialization vector, and update the node_desc file entry for the N_ID node. Activities to be performed:
  - The KS node verifies the HMAC digest from the received ***nksk_key_req*** frame and then decrypts the payload of that frame using the NTAG field from the node’s N_ID description as the key in both actions.
  - The KS node generates the NKSK and NKSKsign keys, as well as the NKSKiv vector. It then prepares the node’s N_ID description. After encrypting the payload of this description with the NK and NKiv keys and determining the HMAC from NKsign, it saves the updated entry in the local node_desc file. Description of the way to modify the entry fields (only the N_ID field is not encrypted):
    N_ID and NTAG—remain unchanged,
    NKSK, NKSKiv and NKSKsign—generated by KS node,
    TOPIC2 = TOPIC2 field from ***nksk_key_req*** frame.
  - The KS node sends back a confirmation of node registration (***nksk_key_ans*** frame Figure 20) using the TOPIC2 from the received frame. The confirmation frame contains the fields NKSK, NKSKiv, NKSKsign, and TOPIC1. The frame payload is encrypted using the registered node’s NTAG. HMAC is also determined, using the registered node’s NTAG, for concatenating the explicit part of the frame and the result of the encryption.
(3) **Acquire NKSK, NKSKiv, NKSKsign and TOPIC1.** Activities to be performed:
  - The N node verifies the HMAC digest from the received ***nksk_key_ans*** frame, and then, it decrypts the payload of that frame using the node as the key in both actions.
  - Save the received data in NVRAM of TPM.

**Figure 21:**
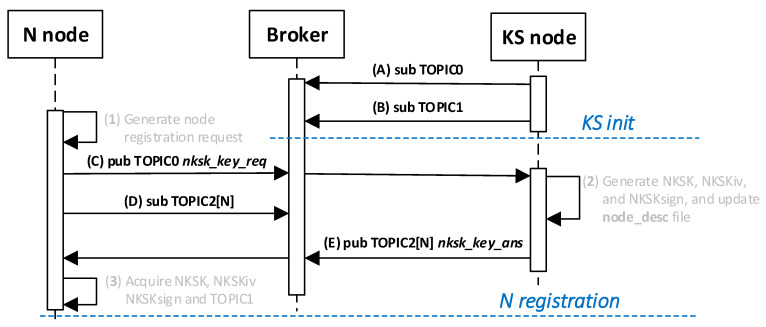
MQTT service data exchange diagram for the N node registration procedure (taken from [21]).

Figure 21 shows how the MQTT service resembles the data exchange during the node registration procedure N.

#### 3.8.8. Establishing Cooperation N Node with the Broker Node

The procedure starts the cooperation of the N node with the Broker node and starts subscription in the topic “TOPIC2”. After completing this procedure, the status of the N node is set to “WORK” in the RN variable.

#### 3.8.9. The Procedure for Generating and Distributing Symmetric Keys

Paper [23] describes, in detail, the generation and distribution of symmetric keys in the KGRD system. Here, only general information about this procedure is presented.

This procedure aims to generate and securely distribute new cryptographic material for a pair of N-type nodes (N1 and N2 will be used to denote the elements of a pair of nodes). The material includes an NNSK (*Node to Node Security Key*) and an initialization vector for this key to encrypt the exchanged data between the pair of N1 and N2 nodes, as well as an NNSKsign (*Node to Node Security Key for Signing*) key to determine the HMAC for the exchanged data.

**Figure 22:**
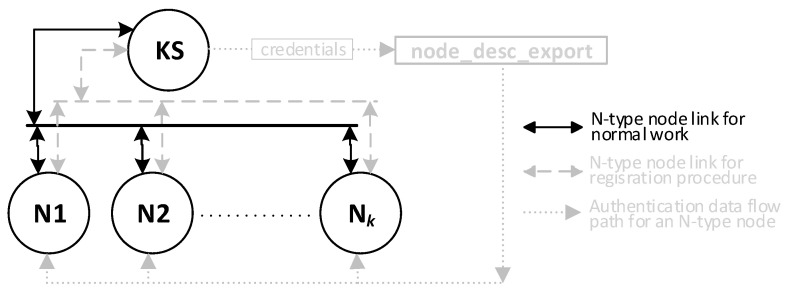
The interaction of N nodes with the KS node when generating and distributing keys (adapted from [21]).

**Figure 23:**
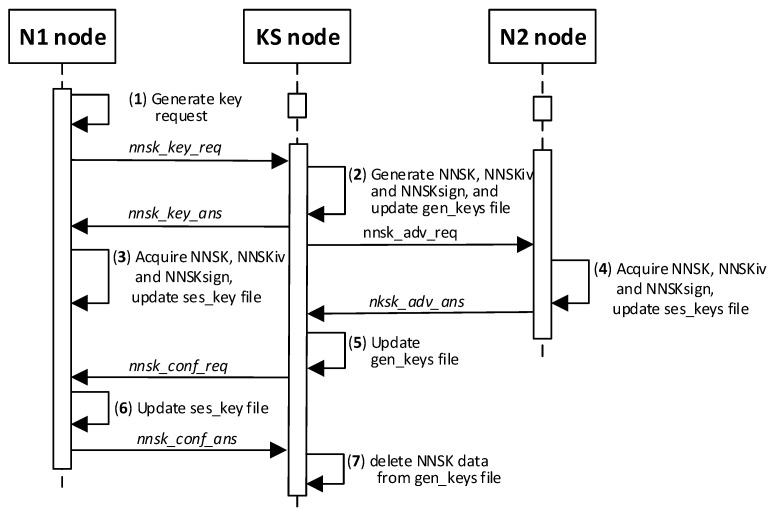
The sequence diagram for the symmetric key generating and distributing procedure (taken from [21,23]).

The procedure result will be the mentioned cryptographic material and the TOPIC3 and TOPIC4 topics, which the KS node will securely forward to N1 and N2 nodes, respectively. N1 and N2 nodes will use these topics in the MQTT service for secure data exchanges. The exchanged data will be secured using generated cryptographic material. Figure 22 shows how the N node interacts with the KS node during the generation and distribution of keys. Figure 23 shows the sequence diagram for generating and distributing keys.

**Figure 24:**
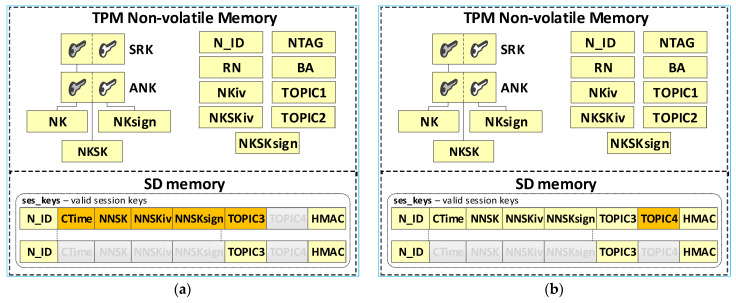
The contents of the resources of node N after step (3) (**a**) and after step (6) (**b**) of the procedure for generating cryptographic material for the first cooperator of node N (adapted from [21]).

During the procedure, the contents of the ses_keys file stored in the SD memory of node N change. Figure 24a shows the contents of this file after step (3) (the data updated so far are highlighted in yellow, and the updated data after step (3) are highlighted in orange). Figure 24b shows this file after step (6) of the procedure (the updated data after step (6) are highlighted in orange).

#### 3.8.10. Secure Data Exchange between N-Type Nodes

**Figure 25:**
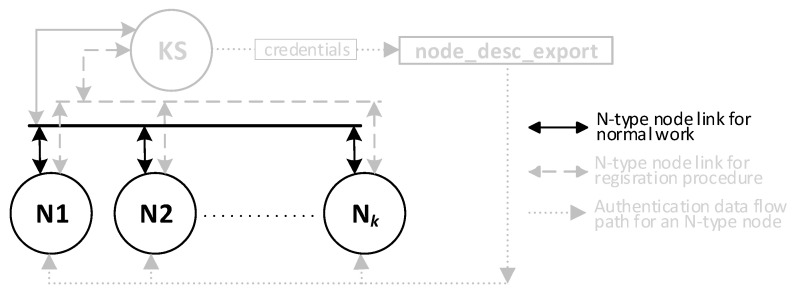
The interaction of the N1 node with the N2 node during data exchange (adapted from [21]).

**Figure 26:**
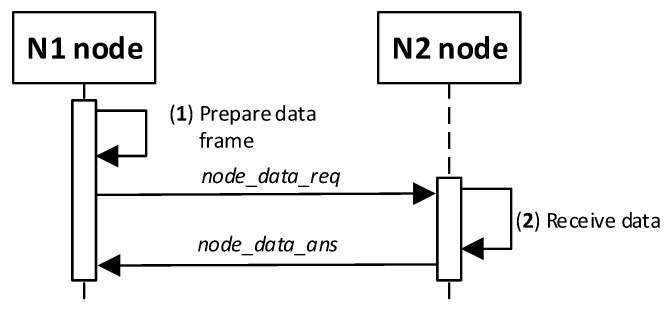
Diagram of the sequence of sending a data packet from node N1 to node N2 (taken from [21]).

The purpose of the KGRD system is to generate and securely distribute cryptographic material for securing data exchanges between two N-type nodes. Such an effect can be obtained after successfully executing the procedure described in the preceding section. How the two nodes will use the generated keys strictly depends on the implementation of the data exchange method between the nodes. This issue is not the subject of this paper. To demonstrate the KGRD system operation, I assumed that node N1 would send a 12-byte string to node N2 and wait to acknowledge the receipt of the transmitted data. Figure 25 shows how nodes N1 and N2 interact during the described data exchange. Figure 26 shows the sequence diagram for this experiment.

The following are the descriptions of both procedure stages.

**Figure 27:**
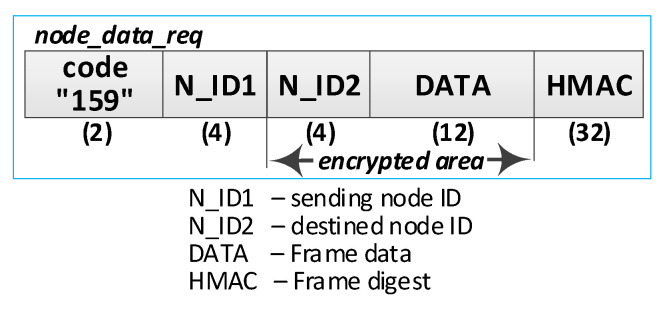
The data frame N2 (taken from [21]).

**Figure 28:**
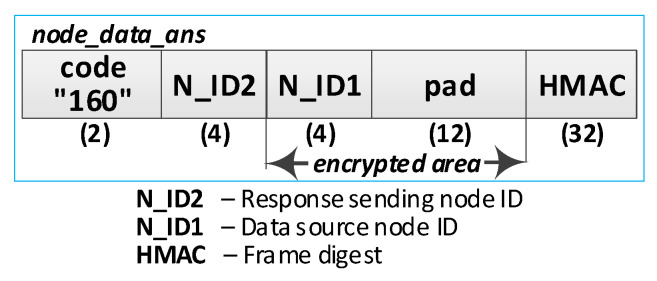
Confirmation frame for receiving data N2 (taken from [21]).

(1) **Prepare data frame.** Activities to be performed:
  - The N_ID1 node prepares the ***node_data_req*** frame (Figure 27). The frame contains N_ID1 (identifier of the node sending the data), N_ID2 (data recipient identifier), and DATA fields. The N_ID1 node encrypts the N_ID2 and DATA fields, using an NNSK and NNSKiv key known only to both nodes, and determines the HMAC for all areas in the frame using the NNSKsign key that is also known only to them;
  - the N node sends the ***nksk_data_req*** frame using TOPIC3 topic. The N_ID2 node subscribes to this topic to receive data from the N_ID1 node.
(2) **Receive data.** Activities to be performed:
  - The N_ID2 node first verifies the HMAC digest from the received ***node_data_req*** frame, and then, it decrypts the payload of that frame using the NNSK and NNSKiv fields generated for N_ID1 and N_ID2 nodes;
  - the N_ID2 node extracts data from the received frame;
  - the N_ID2 node sends back a confirmation of the data frame (***node_data_ans*** frame Figure 28) using the TOPIC3 subscribed by the N_ID1 node. The N_ID2 encrypts the field N_ID1 using the NNSK and NNSKiv, and it determines HMAC using NKSKsign.

**Figure 29:**
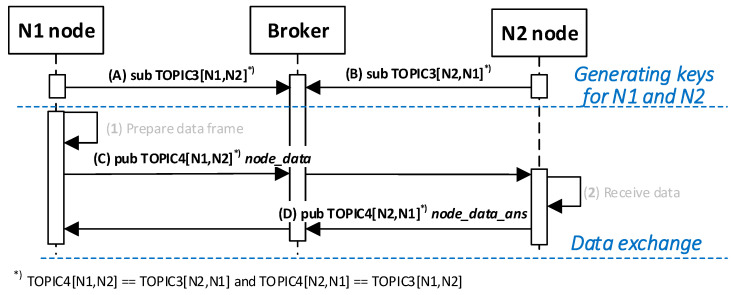
MQTT service data exchange diagram for data transfer from N_ID1 to the N_ID2 node (adapted from [21]).

Figure 29 shows how the MQTT service looks similar to the data transfer from N_ID1 to the N_ID2 node.

#### 3.8.11. The Procedure for Renewing the Keys

Every cryptographic key ages and has to be renewed from time to time. Without key renewal, the risk of that key being guessed increases. Various measures can be used to assess the validity of a key, including the time that has passed since it was generated. The CTime field (stored in the **sys_keys** file in the description of each key used) can be used to determine the expiration time of a given key. Another measure can be the volume of data that has been encrypted with a given key. The presented system does not support any such tasks, but it allows for the renewal of symmetric cryptographic keys.

For the KGRD system, the procedure for renewing keys and generating new keys is the same because the KGRD system does not store the history of generated keys. Therefore, when one node of a pair decides that the key currently in use has expired, it removes the old cryptographic material from its ses_keys file and initiates the key generation procedure for that pair. When receiving a message about generating a new set of keys for that pair (the ***nnsk_adv_req*** frame), the second node of the pair also removes the old cryptographic material from its ses_keys file.

#### 3.8.12. Procedure for Restarting the Node after Powering on Again

The software TPM is used in the KGRD system as a cryptographic coprocessor. The cryptographic material of each node necessary for its operation is stored in the resources of the hardware TPM. The keys needed for encryption before performing this operation are transferred to the trust structure in the software TPM using a particular secure procedure, involving duplicating the required keys in the hardware TPM and importing this key into the software TPM.

Due to the security requirements for the KGRD system and the characteristics of the software TPM, the data stored in the software TPM is lost every time the power is turned off. This situation forces the trust structure in the software TPM to be re-generated after each power-up, and the keys required for encryption are transferred from the hardware TPM resources to the software TPM. The described situation occurs in the following cases:

- after powering off the KS node, when this node is in one of the states {INIT_FULL, READY, WORK};
- after powering off the N node, when this node is in one of the states {INIT_FULL, KWN_COOP, REGISTERED, WORK}.

In both cases, the procedure for restarting the node after powering it on again involves two steps:

1. Generate a temporary trust structure in a software TPM, including CCRK and CCK keys.
2. Duplicate the NK key from the hardware TPM module and securely import this key (NK_v) into the trust structure in the software TPM module.

**Figure 30:**
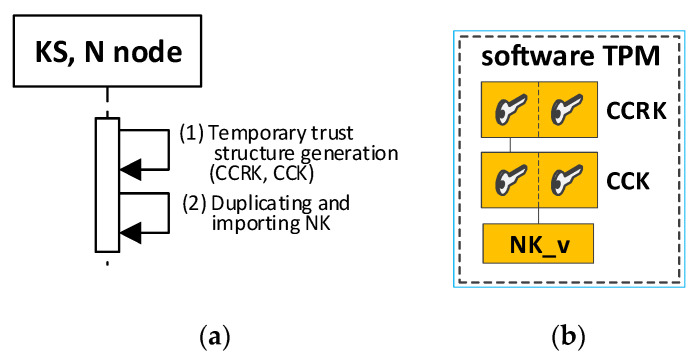
Sequence diagram (**a**) and TPM’s software resources (**b**) after the procedure for restarting the KS and N nodes after powering on again.

Figure 30 shows the course of action during the restart of KS and N nodes after powering on again, as well as the contents of the software TPM resources of these nodes. The hardware TPM resources remain unchanged after this procedure (the created data after restarting the node are highlighted in orange).

After this procedure is completed, the node’s status is set to the status value set before the node is powered off.

### 3.9. Security Evaluation of KGRD System Solutions

Solutions used to secure the Internet of Things should include preventive, detecting, and reactive means [24]. The KGRD system focuses on preventative measures, which does not mean that the system has wholly abandoned detective and reactive measures. Preventive actions include solutions to prevent or hinder the successful execution of typical attacks.

The primary purpose of the KGRD system is to generate cryptographic keys for data exchange between clusters of IoT network nodes. Such groups of nodes usually use wireless connectivity. These nodes are often unattended and usually become easy targets for attack. Given such conditions, the following attacks will be considered: installing a fake sensor node (node replication attack), impersonating valid sensor nodes (imitation), attacks on transmitted data, DoS attacks, attacks on the routing process [24], and botnet activities. The solutions presented in the KGRD system are designed to prevent these attacks. The mechanisms offered by the TPM are the basis for these solutions. These mechanisms make it possible to secure cryptographic keys by building key hierarchies and local trust structures, cryptographically protecting data stored in the sensor node’s resources (in the TPM’s NVRAM and SD memory), and maintaining the integrity of transmitted and stored data through HMAC hashing. The following subsections present ways to counter the attacks mentioned above and describe which TPM mechanisms were used for this purpose.

#### 3.9.1. System Reboot or System Crash

The credentials of each node are stored in the protected resources of the hardware TPM all the time. Powering down (rebooting the system) does not result in the loss of this data. After rebooting the system, the procedures must be repeated. Before repeating these procedures, you must delete the residual data remaining after unfinished procedures.

A critical component of the KGRD system is the KS node—specifically, its local trust structure and the data stored in the TPM’s NVRAM and SD memory. The SRK key, stored in the TPM hardware resource, is the basis of data security. This key is placed at the top of the trust hierarchy, and its private part is inaccessible outside the TPM module. To increase the level of security and the system’s resilience to failures, a backup of the cryptographic material stored in the system can and should be prepared. Preparing such a backup involves duplicating part of the key hierarchy, starting with the ANK key.

The duplication procedure is very similar to the procedure for exporting keys from a hardware TPM to a software TPM, which is described in Section 3.5. The difference is that the new ANK key parent comes from another hardware TPM. Once the key hierarchy is exported, the TPM’s NVRAM and SD data can be straightforwardly copied from one system to another because duplicate keys protect them.

This approach allows the system (backup) from which the new ANK key parent is derived to be easily used in case the original KGRD system fails. The only item that would need to be updated in the backup system, after a failure of the original system, is the contents of the files stored in SD memory, which is done by simply copying them.

The occurrence of a failure (reboot) of the KS node during the N node registration, key generation, or renewal procedures will result in an erroneous termination of these procedures for N nodes. However, there is nothing to prevent the N node from repeating such a procedure after the KS node has started.

There are two cases to consider if an N-type node fails and needs to run on another machine. Suppose the N node has previously completed the registration procedure, and a backup of its cryptographic material has been created on another node. In that case, this other node, from the point of view of the KGRD system, can immediately take action. If the given conditions have not been met, the new node should obtain the credentials from the AC node and start the registration procedure. Once this procedure is completed, the node will be ready for operation.

Performing the described steps allows you to make the KGRD system immune to the various failures that various attacks can cause.

#### 3.9.2. Node Replication Attack

The attack is based on adding a copy of another node to a running network. The lack of security and supervision of the node creates the conditions for replication attacks to be efficiently executed. Launching such an attack with all the nodes in the KGRD system is extremely difficult. Each node in the KGRD system has a hardware TPM installed. A hierarchy of keys in its resources creates a local trust structure. Each key hierarchy begins with an asymmetric SRK key, which is generated only once and is non-removable. The TPM stores the private part of this key in the internal structures of the module. This part of the key is inaccessible for reading. This key secures all other keys used by the node, which are used to secure the node’s sensitive data.

If an adversary tried to build a copy of the original node, it would have to equip that copy with another copy of the hardware TPM module. This other TPM module cannot generate an identical trust structure to the original node. The SRK key of such a copy, which is at the top of its hierarchy, will undoubtedly differ from the original one. This different trust structure will make it impossible to read the original data, which is cryptographically secured.

The KS node is initialised in a secure and supervised area, and once it is fully prepared, it can start serving N nodes. The KS node generates authenticating data for other nodes of the system. These data go to new N nodes, via the AC node, using a secure procedure that is executed in a controlled area. The authenticating data for the N node is the basis for registering the new node in the KGRD system. Only registered N nodes can request new keys from the KS node.

Even if the adversary succeeded in intercepting the credentials intended for a node at the preparation stage of that node, and this valid node registers with the KS node’s resources earlier than the fake node, the acquired credentials will already be useless. The KS node will reject all attempts to register a node already registered. The only chance for the adversary is to get ahead of the valid node in the registration procedure, which is difficult because the valid node will start this procedure earlier.

N-type nodes of the KGRD system are most vulnerable to attacks in the preparation phase before they are registered. For this reason, an important consideration is a need to perform these activities in a secure and supervised environment outside the nodes’ normal operating area.

An adversary may use brute force to take over the data stored in the node’s resources. The TPM module’s Platform Configuration Register (PCR) can be used to prevent such an action, as it allows for the detection of and response to such unauthorised actions. It is possible to detect such activities using a mechanism constructed on the PCR registry. The use of the PCR registry is not the subject of this paper.

#### 3.9.3. Sensor Impersonation

Trying to impersonate a proper node is challenging to do. When preparing a node, the AC transmits its N_ID and the NTAG tag specially formulated for it to the node. At this point, the node learns what identifier (N_ID) it will have. The NTAG tag is determined for each N node by the KS node. The content of the NTAG tag is a SHA256 hash designated for the string, which is the concatenation of three fields. These fields include:

- N_ID of the node;
- the private part of the ANK key;
- the 4 byte area containing the number of the entry about the description of this node in the node_desc file.

The private part of the ANK key is known only to the KS node, and only a valid KS node can verify the correctness of the NTAG tag. During node N’s registry procedure, the KS node verifies the NTAG tag’s correctness. This approach gives confidence to the KS node that it is registering the node N for which it generated this tag. In turn, it gives confidence to the N node that the correct KS node is registering it. Entangling the sequence number of the node’s description in the NTAG tag makes it even more difficult for the adversary to generate a false NTAG tag based on other captured NTAG tags because this sequence number is known only by the KS node.

During the registration procedure, the KS nodes and the registered node N establish shared symmetric keys (NSK, NSKsign) to secure a future data exchange. An impersonating node is unable to take over the registered node’s data.

TPM stores the node identifier N_ID, tag NTAG, and shared keys NSK and NSKsign in its secure resources. The NTAG tag is critical in countering this attack. This tag is only stored and transferred in a secured form from the moment it is generated.

#### 3.9.4. Attack on Transmitted Data

Data transmitted over wireless networks are vulnerable to eavesdropping, traffic analysis, injection of other data, modification, and transmission interruption. The KGRD system uses node authentication. The KGRD system uses the Encrypt-then-MAC (EtM) method to ensure the confidentiality and data integrity of all stored and transferred data. This approach requires two keys, known only to the individual pairs of nodes exchanging data, to secure the transmitted data. In the KGRD system, a different key pair is generated for each pair of nodes exchanging data to apply the EtM method. The first key is used to encrypt the data (confidentiality), and the other is used to determine the HMAC digest (integrity).

As an additional security feature, the system generates random strings for topics used by the MQTT service. The mechanisms of the KGRD system ensure that only the parties that exchange data will know the content of these topics. The KGRD system distributes these topics simultaneously with the distribution of cryptographic keys.

#### 3.9.5. Denial of Service

The group of DoS attacks is numerous. An adversary can execute these attacks in the physical and upper layers. The solutions used in the KGRD system do not address this type of attack. It is recommended to use known methods to protect KS and N nodes from this attack independently of the KGRD system.

#### 3.9.6. Routing Attacks

Typical targets of attacks in wireless networks are the routing protocols in use. Such attacks include Sinkhole Attacks, Wormhole Attacks, False Routing Information, Sybil attacks, and Selective Forwarding. These attacks require placing a fake node on the network or manipulating the operation of selected nodes. In a KGRD system environment, the ability to successfully execute such an attack is minimal. Section 3.9.2 and Section 3.9.3 describe the mechanisms that counter these attacks.

#### 3.9.7. Botnet Activities

Significant threats to IoT networks are botnets. Botnets spread an infection to misconfigured nodes as a first step, and then, the infected nodes attack the selected target after the party managing the bot issues the appropriate command. The Mirai malware is an example of such a bot [25]. The PCR registry capabilities of the TPM module make it possible to protect against bot injection, but this solution is not part of the presented KGRD system.

## 4. Results

### 4.1. Demonstrator

A demonstrator has been set up to check the operation of the KGRD system. When building the demonstrator, I assumed it would include one KS-type node, two N-type nodes (N1 and N2), and a node that would be an MQTT service broker (Broker). The demonstrator does not have an AC node. In its place, a software package was prepared that performed the following tasks:

- Exporting these credentials for N nodes to an external node_desc_export file—on the KS node, export activity extended the function of preparing the credential data for N nodes.
- Importing credentials for an initialised node N—an initialised node N imports one set of the credential data from the node_desc_export file before starting the procedure for initializing that node.
- Setting the list of cooperators of the node being initialized—the initialised node N obtains a list of N node identifiers from the node_desc_export file.

Preparing the demonstrator was necessary to demonstrate the correctness of secure key generation and distribution. The steps involved in passing credentials to N nodes before initialization and registration are critical to system security. The author of the solution is aware that the solution described above, which replaces the performance of AC node tasks in the system, does not meet all security requirements. However, these activities do not directly affect the key generation and distribution process.

**Figure 31:**
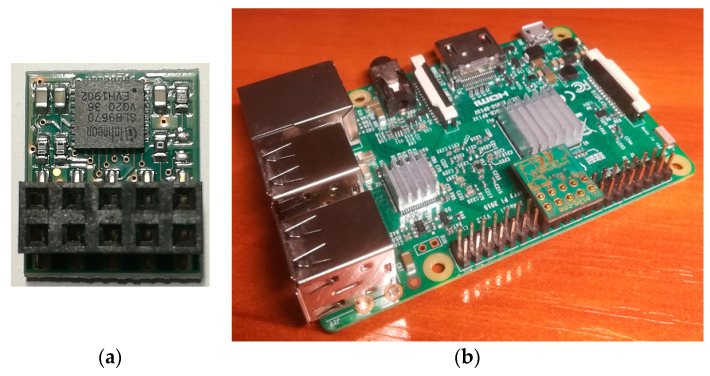
View of the Infineon Optiga™ SLB 9670 TPM 2.0 module (**a**) and how the TPM module was installed on the Raspberry Pi board (**b**).

**Figure 32:**
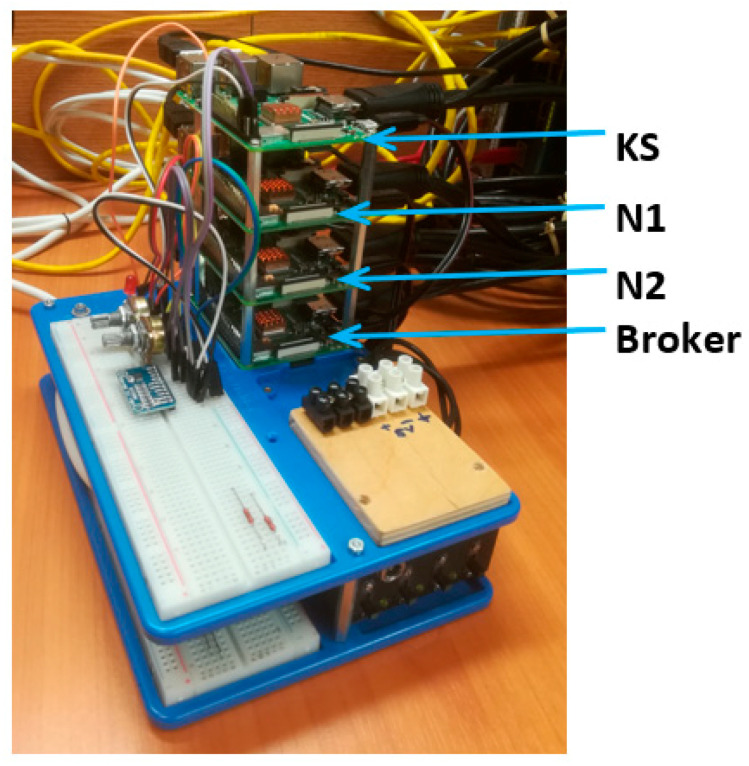
A view of the KGRD system demonstrator (taken from [23]).

Raspberry Pi Model B boards with 32 GB SD memory are the base of all four nodes of the developed demonstrator. All nodes have a hardware TPM module installed (LetsTrust TPM with Infineon Optiga™ SLB 9670 TPM 2.0 chip). Figure 31 shows a view of the TPM module and how the TPM module was installed on the Raspberry Pi board. The broker uses Mosquitto 1.5.1 software to support the MQTT 3.1.1 protocol. An Ethernet interface was used to exchange data between the demonstrator nodes. Figure 32 shows a view of the demonstrator.

The MQTT service uses the connection-oriented TCP protocol to send messages between the broker and the other nodes in the transport layer. Still, from the point of view of message transmission, this is a stateless protocol. Therefore, a simplified control mechanism was introduced for both sides of the data exchange in the KGRD system to have control over the state of the data exchange protocol. This mechanism is that each node, during data exchange, after each sending of a message, prepares its environment to handle only one message from the cooperating node among the messages expected in a given context. For example, node N1, after sending the ***nnsk_key_req*** frame at the beginning of the key generation and distribution procedure, will wait for only one ***nnsk_key_ans*** message from node KS. This approach allows for control over the system’s data exchange protocol, but it can handle only one pair of N1 and N2 nodes simultaneously. It will be ready to handle the next pair after it has finished handling this pair of N nodes. The mechanism used is a particular shortcoming of the demonstrator’s implementation, which does not interfere with the validity of the KGRD system’s solutions.

### 4.2. Test Cases

**Figure 33:**
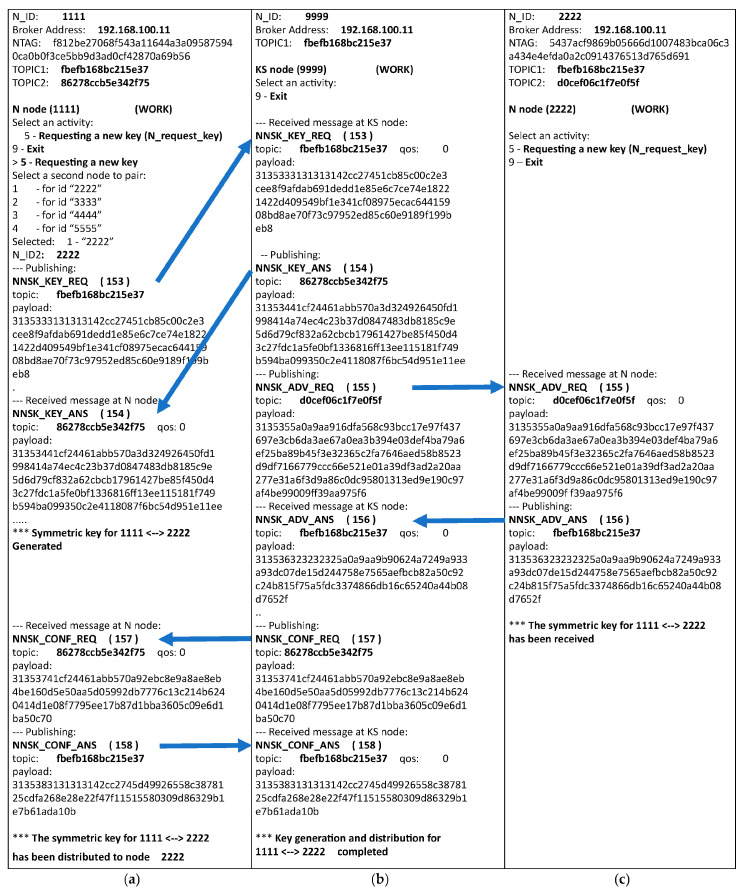
Node console view of N1 (**a**), KS (**b**), and N2 (**c**) during key generation and distribution for N1 and N2 nodes.

Test scenarios were developed for all 12 procedures of the KGRD system described in Section 3.8, and the system’s operability was verified using them. The most interesting test case for the KGRD system functions is the generation and distribution of cryptographic keys. This test case corresponds to a procedure with a similar name, a detailed description of which you can find in Section 3.8.9. Figure 33 shows the flow of this test as observed on the consoles of the N1 (a), KS (b), and N1 (c) nodes. In this figure, the arrows show the transfer of successive frames sent between the nodes participating in the data exchange. Note the random content of the topics used and the encrypted content of the frames sent.

**Figure 34:**
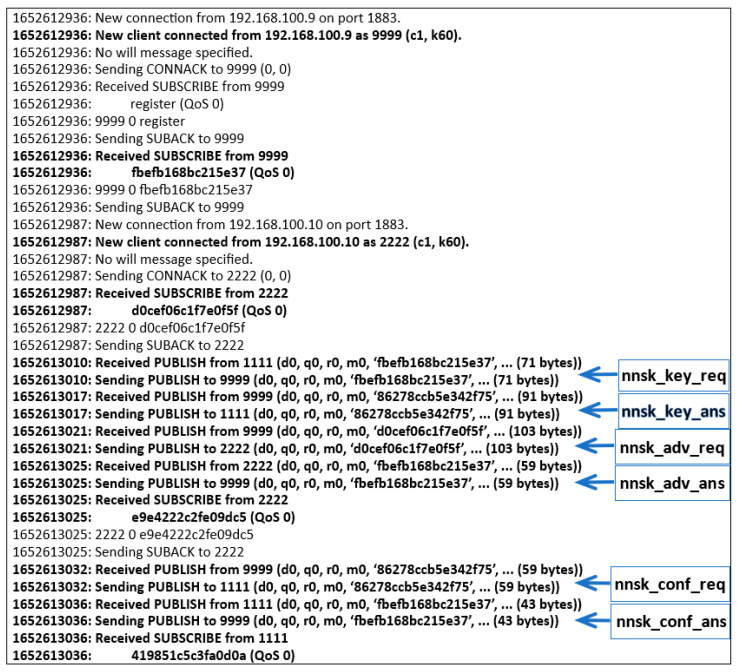
The transfer of messages on the MQTT server during the generation and distribution of keys for nodes N1 and N2.

Figure 34 illustrates the transfer of messages on the MQTT server during the generation and distribution of keys for nodes N1 and N2. Note the content of the topics in the following messages.

From the data shown in Figure 34, it is possible to deduce the time required to execute the key generation and distribution steps (the first column of each record gives the time moment of the start of a given step). Table 3 shows the duration of the selected steps of this procedure. The execution time per step includes the activities performed by the node related to the generation and distribution of keys, as well as the activities related to the preparation and reception of the MQTT message.

Table 4 shows the durations of selected steps of other KGRD system procedures. The data in this table were obtained in other experiments that are not described in the paper. Some items in the table are noteworthy, with times reaching tens of seconds. These items concern situations for generating asymmetric cryptographic keys or cryptographic operations using asymmetric keys. The hardware TPM module performs all of these operations. Fortunately, these operations in the system are performed once. The generation and security of symmetric keys do not exceed 1 s, which is the most crucial parameter for the KGRD system.

## 5. Discussion

For each system procedure, test scenarios were prepared. The scenarios were used to check the operation of the KGRD system. The results of these tests confirmed the correct operation of the KGRD system. The previous chapter only presents selected results that illustrate the system’s operation during key generation and distribution. The results presented here show that the data sent in each step of the procedure are not in an explicit form. This observation is particularly relevant for the content of the frames sent and the topics used.

Table 4 shows selected data on the time required to perform selected tasks. All these periods are given in seconds. It might seem to be a very long time. Using the MQTT service to exchange data considerably impacts the size of this time. It is also significant that each of these tasks requires multiple cryptographic operations. Depending on the task, the operations may include obtaining keys and initialization vectors for these operations from cryptographically secure locations, symmetric or asymmetric encryption, and HMAC hash determination. The most significant values occur in procedures for creating local trust structures. These operations require the generation of several asymmetric keys and, despite hardware support by the TPM, take tens of seconds. The good news, in this context, is that the most time-consuming steps are performed once. The demonstrator uses an Ethernet interface with a high bandwidth compared to the weak links used by IoT network nodes. Indeed, the use of such links will increase these times. However, it is worth noting that a key acquisition time of a few or several seconds for IoT devices is not a critical value. What is essential for these devices is ensuring security and the short transfer time of secured data, which did not exceed 2 s in the experiments.

The AC node’s tasks have been replaced by special procedures, described in Section 3.8.3 and Section 3.8.5, to simplify the demonstrator’s implementation. Preparation of the implementation of the original AC node will be the subject of future work.

The basis of the solution presented in the article is a hardware TPM v.2.0 module. The use of this module provides a number of capabilities that significantly impacted the design of the solution. These properties of the module include:

- hardware generation of asymmetric and symmetric cryptographic keys;
- hardware generation of random numbers with high entropy;
- secure storage of cryptographic material of the internal NVRAM of the TPM;
- creating key hierarchies that can be used to build local trust structures;
- hardware support for encryption/decryption;
- support for Platform Configuration Registers that can be used for protection and attestation; internal node resources (this property was not used in the presented study).

The effect of the hardware implementation of the TPM module is that it has some limitations. These limitations include:

- a limited list of supported asymmetric algorithms—RSA2048, RSA3072, RSA16384, ECC256, ECC384, and ECC521 are available;
- a limited list of supported symmetric algorithms—3DES, AES128, and AES256 are available;
- a limited list of supported hash functions—SHA-1, SHA256, SHA384, SHA512, and HMAC are available;
- the ability to generate key hierarchies based on RSA keys only;
- some hardware implementations do not support certain functions, e.g., encryption and decryption for symmetric algorithms. This problem was essential to implementing the solution to the previously published solution concept.

There are many solutions for lightweight cryptographic algorithms, the characteristics of which can be found in [26]. The solution demonstrator selects those supported by the TPM v.2.0 module and meets NIST [27] requirements. These include RSA-2048, AES-128, SHA-256, and HMAC using SHA-256.

A limited number of writes characterise NVRAMs. In the case of the NVRAM of the TPM module, this will not be a problem because this memory is only uprooted for writing during the preparation and registration phase of each node (both KS and N types). Then, the number of writes will not exceed 100 in each TPM module. During regular system operation, only the contents of the NKSKiv vector on N nodes will require writes, as the other fields will only be read. The NKiv vector will be read-only. If some entries from a file in SD memory need to be encrypted/decrypted, an initialization vector will be needed. The contents of NKiv, N_ID, and the position of this entry in the file determine this vector.

The most sensitive part of the system is the procedure for preparing a node until it is registered. At each node’s life stage, it would be easiest for an adversary to defeat the system’s safeguards. Therefore, these activities should be performed in a protected and controlled environment outside the area of the regular operation of system components. Section 3.9.2 presents the details.

As presented, the KGRD system is not equipped with mechanisms to counter DoS-type attacks. Mechanisms similar to those used in traditional IT systems to increase the system’s resistance to such attacks can be used.

A hardware TPM module makes it possible to use its PCR register to construct a detecting and notifying mechanism for unauthorised tampering with the hardware and software configuration resources of each KGRD system node. Using such a solution will immunise the system nodes, which can be placed in physically weakly protected areas. Such a solution will be the second task in future work.

## Figures and Tables

**Table 1:** Purposes of topics in the KGRD system (adapted from [21,23]).

Topic	Node	Purpose
TOPIC0 ^1^	KS	An N-type node will use this topic to send the first request in the registration procedure
TOPIC1	KS	An N-type node will use this topic to send subsequent requests in the registration procedure
TOPIC2	N	KS node will use this topic to send messages to N-type nodes
TOPIC3*_mn_*	N*_m_*	The N*_m_* node will use this topic to receive messages published by the N*_n_* node
TOPIC4*_mn_*	N*_m_*	The N*_m_* node will use this topic to publish messages to the N*_n_* node

^1^ TOPIC0 has “register” content, and the others have randomly generated content.

**Table 2:** Descriptions of the states of KS and N-type nodes.

State Name	Node Type	Description	Conditions That Must Be Met
NOT_INIT	KS, N	node is not initialized	The trust structures for the node and the required data have not been created in the NVRAM of the TPM
INIT_FULL	KS, N	node initiated in full	The trust structure is generated in the hardware TPM
			The required data is generated in the NVRAM of the hardware TPM
			The trust structure is generated in the software TPM
INIT	KS, N	node initialized after powering on again	The trust structure is generated in the hardware TPM
			The required data is generated in the NVRAM of the hardware TPM
			LACK of trust structure in the software TPM
READY	KS	ready to register N nodes	KS node is fully initiated
			File “node_desc” is generated
			forwarded credentials for nodes N
REGISTERED	N	node N registered	N node is fully initiated
			There is an NKSK key in the trust structure in the hardware TPM
			In the NVRAM of the hardware TPM, the existence of fields NKSKiv, NKSKsign and TOPIC1
			There is an NKSK_v key in the trust structure in the software TPM
WORK	KS	ready for normal operation	KS node is ready to register N nodes
			Communication with the MQTT broker is up and running
WORK	N	ready for normal operation	N node is registered
			Communication with the MQTT broker is up and running
KWN_COOP	N	known cooperators	N node is fully initiated
			File “ses_keys” is generated
INIT_REG	N	After power on again, when the N node was previously in a REGISTERED state	Forwarded credentials for nodes N
			There is an NKSK key in the trust structure in the hardware TPM
			In the NVRAM of the hardware TPM, the existence of fields NKSKiv, NKSKsign and TOPIC1

**Table 3:** The durations of selected steps of the key generation and distribution procedure.

Operation	Number ^1^	Time [s]
Generation NNSK, NNSKiv and NNSKsign with the gen_keys file support (on the KS node)	(2)	**7**
Receiving NNSK, NNSKiv, NNSKsign with the ses_keys file support (on the N2 node)	(4)	**4**
Update the gen_keys file (on the KS node)	(5)	**7**
Support of ses_key file (on the N1 node)	(6)	**4**

^1^ The number of the stage shown in Figure 23.

**Table 4:** The durations of selected steps from selected KGRD system procedures.

Procedure	Operation	Number	Time [s]
The KS node initialization procedure ^1^	Generating local trust structure (SRK, ANK)	(1)	**40–60**
	Generate keys NK and NKsign	(2)	**4**
	Generating a temporary trust structure (CCRK, CCK)	(3)	**15–20**
	Duplicating and importing NK	(4)	**8–10**
	Generate N_ID, TOPIC1 and NK_iv and set BA address	(5), (6), (7)	**5–7**
The N node initialization procedure ^2^	Generating local trust structure (SRK, ANK)	(1)	**40–60**
	Generate keys NK and NKsign	(2)	**4**
	Generating a temporary trust structure (CCRK, CCK)	(3)	**15–20**
	Duplicating and importing NK	(4)	**8–10**
	Load N_ID and NTAG	(5)	**2**
	Generate NKiv, NKSKiv, NKSKsign, and TOPIC	(6)	**4–6**
	set BA address	(7)	**1**
N node registration procedure ^3^	Generate NKSK, NKSKiv and NKSKsign, and node_desc file support	(2)	**4–5**
Data exchange between nodes N1 and N2 ^4^	Acquire data	(2)	**2**

^1^ The sequence numbers of the stage shown in Figure 10. ^2^ The sequence numbers of the stage shown in Figure 13. ^3^ The sequence number of the stage shown in Figure 17 and Figure 21. ^4^ The sequence number of the stage shown in Figure 26 and Figure 29.

## Data Availability

Data is not publicly available due to the classified nature of the work.
